# Validity of the Italian Version of DIVA-5: Semi-Structured Diagnostic Interview for Adult ADHD Based on the DSM-5 Criteria

**DOI:** 10.3390/healthcare13030244

**Published:** 2025-01-26

**Authors:** Rosaria Di Lorenzo, Emanuela Latella, Federica Gualtieri, Anna Adriani, Paola Ferri, Tommaso Filippini

**Affiliations:** 1 Department Mental Health and Drug Abuse, AUSL-Modena, 41121 Modena, Italy; r.dilorenzo@ausl.mo.it (R.D.L.); e.latella@ausl.mo.it (E.L.); 2 School of Specialization in Psychiatry, Department of Biomedical, Metabolic and Neural Sciences, University of Modena and Reggio Emilia, 41125 Modena, Italy; f.gualtieri@ausl.mo.it; 3 School of Medicine, Department of Biomedical, Metabolic and Neural Sciences, University of Modena and Reggio Emilia, 41125 Modena, Italy; 4 Section of Public Health, Department of Biomedical, Metabolic and Neural Sciences, University of Modena and Reggio Emilia, 41125 Modena, Italy; paola.ferri@unimore.it; 5 Environmental, Genetic and Nutritional Epidemiology Research Center (CREAGEN), Section of Public Health, Department of Biomedical, Metabolic and Neural Sciences, University of Modena and Reggio Emilia, 41125 Modena, Italy; 6 School of Public Health, University of California Berkeley, Berkeley, CA 94704, USA

**Keywords:** adult ADHD, psychometric scales, construct and concurrent validity, factor analysis

## Abstract

**Introduction:** In 2019, an updated version of the Diagnostic Interview for ADHD in Adults (DIVA-5) was developed based on DSM-5 criteria, currently validated in Korean and Farsi. The aim of this study is to validate the DIVA-5 Italian version. **Methods:** 132 subjects in the Adult ADHD Screening Center of AUSL-Modena, who agreed to participate in this study, were selected. Socio-demographic and clinical variables were collected. DIVA-5, Barkley Adult ADHD Rating Scale (BAARS), and Adult ADHD Self Rating Scale (ASRS-v1.1) were administered. We assessed the internal consistency of the DIVA-5 Italian version and its concurrent validity with ASRS-v1.1 and BAARS-IV. An exploratory factor analysis (EFA) was conducted to evaluate the construct validity, and a multiple linear regression to evaluate the predictive validity. **Results:** Our analysis indicated good internal consistence of the DIVA-5 Italian version (Cronbach’s alpha and Kuder coefficients ranged between 0.61 and 0.78). The EFA showed five factors representing specific variance. The correlation between the corresponding ADHD dimensions of DIVA-5 and BAARS was found to be statistically significant (Spearman’s coefficient ranged between 0.61 and 0.47, *p* = 0.000), while the correlation between the DIVA-5 dimensions and ASRS-v1.1 was statistically significant for all the dimensions except child hyperactivity/impulsivity. The multiple linear regression showed a positive association of the DIVA-5 score with the “job” variable and a negative association with “drug therapy”. DIVA-5 showed greater sensitivity for inattention in adulthood and greater specificity for hyperactivity/impulsivity in childhood. **Conclusions:** Our results confirm that the DIVA-5 Italian version represents a valid and reliable tool to diagnose adult ADHD.

## 1. Introduction

Attention deficit hyperactivity disorder (ADHD) is a neurodevelopmental disorder characterized by a persistent pattern of inattention and/or hyperactivity–impulsivity, which onsets before age 12, with a prevalence of 5–7% in childhood [1,2,3] and 2.5% up to 5% in adulthood [4,5,6,7]. In 2023, an estimated 15.5 million U.S. adults (6.0%) had a current ADHD diagnosis based on self-report; approximately one half received the diagnosis at age ≥ 18 years [8].

To diagnose ADHD, inattention and hyperactivity–impulsivity must be beyond the normal range expected for the age, must be clearly observable in more than one context, and must significantly interfere with academic, occupational, or social functioning [9].

In the child population, ADHD is more common in males, with a 2:1 ratio [3,4,10]. In contrast, the sex ratio tends to balance out in adults [11], suggesting that adult females are diagnosed more often than males [11,12].

The deficit in impulse control and attention typical of ADHD leads to difficulties in many social skills, with problems at school, antisocial behavior, substance abuse, accidents, unemployment, and job and relationship instability [10]. In particular, substance use in subjects with ADHD would serve as “self-medication” to improve mood and sleep, and reduce symptoms [13,14]. Cannabinoids, stimulants, and nicotine are the substances most commonly used by subjects with ADHD [15,16,17,18,19].

Comorbidity with another psychiatric disorder is usually present in both children and adults with ADHD, in about 75% of cases [20,21,22]. In adults, the most frequent concurrent disorders are substance/alcohol dependence and antisocial behavior, followed by anxiety and depressive disorders [23]. In all cases, the presence of ADHD can represent a risk factor for the development of other psychiatric disorders in adulthood, especially in the case of early-onset ADHD with severe symptoms and scarce response to treatments [21,22,23,24].

In the scientific literature, ADHD is no longer considered a purely childhood disorder, since it shows a persistence range between 30% and 60% in adulthood [25,26,27]: some cases of ADHD could in fact have a late onset [28] or could represent a subthreshold child ADHD, with “masked” symptoms until adulthood, when the demands of life skills increase physiologically and ADHD symptoms become evident [24]. In any case, some authors suggest considering ADHD a continuum of clinical manifestations from childhood to adulthood [29,30], as a chronic disorder that can cause significant impairment in quality of life [8,23,30,31].

Early diagnosis and appropriate treatments can prevent long-term negative consequences according to The National Institute for Health and Clinical Excellence [32]. The diagnosis of ADHD in adults would contribute to improvements in functioning, health-related quality of life and self-esteem, compared to subjects with symptomatic but undiagnosed ADHD [7,33].

During the past 10 years, there has been a greater focus on women and adults with ADHD [34,35,36,37]. In particular, interest in ADHD in adults has increased following the COVID-19 pandemic, due to the increasingly more frequent occurrence of this diagnosis [38,39]. A recent study conducted in Finland highlighted that the potential adverse outcomes of pandemic-associated changes in living conditions could have increased the prevalence of ADHD in the youth population [40].

Although several studies attempted to identify biomarkers, EEG [41], or neuroimaging markers for ADHD [42,43,44,45], nowadays the diagnosis in both childhood and adulthood still remains purely clinical.

The clinical manifestations of the disorder vary over time depending on age groups:

- In preschool age, the maximum degree of hyperactivity is recorded;
- In school age, the symptoms of inattention and impulsivity and a possible reduction in hyperactivity are evident;
- In adulthood, the prevalent clinical characteristics are inattention and organizational difficulties, while hyperactivity and impulsivity become less visible with increasing age [46].

The clinical presentation of ADHD with inattention is more common in females [11].

Compared to the child population, fewer diagnostic tests are available for adults.

The most widely used self-report scales are the Adult ADHD Self Rating Scale (ASRS-v1.1) [47,48,49], the Barkley Adult ADHD Rating Scale (BAARS) [50], and the Conner’s Adult ADHD Rating Scale Self-Report Screening Version (CAARS-S-SV), [51], to which the more recent ADHD-SCL-90-R Screening Scale has been added [52].

The Diagnostic Interview for ADHD in Adults (DIVA) is a semi-structured diagnostic interview developed in 2010 by the DIVA Foundation in accordance with the DSM-IV criteria for ADHD in adults, DIVA 2 [53,54].

A revised version, DIVA-5, was developed according to the DSM-5 criteria in 2019 [24]: the criterion of onset age was changed from “before 7 years of age” to “before 12 years of age”; the cut-off for the number of symptoms needed to make a diagnosis of ADHD in adulthood was lowered from six to five symptoms in both the domains of inattention and/or hyperactivity/impulsivity; and the subtypes were redefined as “clinical presentations” and not “subtypes”, as reported by the DSM-IV, which did not show being stable in the disorder course [24].

DIVA-5 has been translated into many languages, but only two of its translated versions have been validated: the Korean-language version [55] and the Farsi-language version [56]. The first study showed that DIVA-5 presented a diagnostic accuracy of 92%, a sensitivity of 91.30%, and a specificity of 93.62% [55]; the second one showed a diagnostic agreement of 81.66% between DIVA-5/SCID-5 diagnoses, 80% between SCID-5/CAARS-S-SV, and 71.66% between DIVA-5/CAARS-SSV, with good to excellent reliability [56].

In recent studies, DIVA-5 showed good reliability in diagnosing ADHD among people with comorbid disorders, such as Substance Use Disorders (SUD) [57], Bipolar Disorders (BP), and Major Depressive Disorders (MMD) [58], and in identifying specific dimensions of impulsivity and emotion dysregulation in behavioral addiction [59].

The primary objective of this study was to validate the Italian version of the DIVA-5 scale; the secondary objective was to report the socio-demographic and clinical characteristics of individuals who were screened for adult ADHD in a psychiatric service.

## 2. Methods

### 2.1. Design, Sample and Variables

The design of this study was single-center, diagnose accuracy investigative, and non-profit. The sample was represented by 132 subjects enrolled at the Adult ADHD Screening Centre of the AUSL of Modena, from 1 August 2023 to 31 May 2024, who met the following inclusion criteria: people over 18 years old, who lived in the Modena area and province, with a suspected diagnosis of adult ADHD, who agreed to participate in this study providing their informed consent.

We selected the following variables of the sample: Socio-demographic: age, sex, nationality, education, employment, marital status, and housing situation. Clinical-anamnestic: family history of psychiatric disorders and/or substance use, psychiatric treatments in childhood and adulthood in psychiatric services and/or with other specialists, pharmacological therapies in childhood and adulthood, psychotherapy, psychoeducational and/or rehabilitative treatments, medical comorbidities, and psychiatric hospitalizations in childhood and adulthood.

### 2.2. Study Procedure

The psychometric scales for ADHD screening suggested by the Emilia-Romagna Regional program of ADHD Centres [60] are represented by the Diagnostic Interview for ADHD in Adults 2 (DIVA-2) [53] and the Adult ADHD Self-Report Scale (ASRS-v1.1). We decided to update the psychometric assessment of ADHD using the more recent DIVA-5 [24], already translated into Italian, but not validated in our language. Therefore, after a clinical interview in a semi-structured form, as in usual clinical practice, to collect socio-demographic and clinical information, we administered the ASRS-v1.1 screener [47,49] and DIVA-5 to individuals of our sample, who had provided their informed consent to participate in this study. For this validation study, we also administered the Barkley Adult ADHD Rating Scale-IV (BAARS-IV) [50], already translated and validated in Italian, in order to compare the results from this scale with those from the other two scales.

### 2.3. Assessment Tools

(1) The Adult ADHD Self-Report Scale (ASRS-v1.1) [47,49] is an 18-item self-report scale based on the DSM-IV symptom criteria developed by the Korean Workgroup on Adult ADHD in conjunction with the World Health Organization [47]. The scale is composed of two parts: parts A and B. Each ASRS question asks respondents how often a particular ADHD symptom had occurred over the past six months using a 5-point Likert scale ranging from 0 (never) to 4 (very often). Part A is called the ASRS Screener, which is the short form of the ASRS, and comprises six questions selected based on a stepwise-logistic regression. Respondents who endorse at least 4 out of 6 items at the ASRS Screener are considered at “elevated” risk for ADHD.

(2) The Barkley Adult ADHD Rating Scale-IV (BAARS-IV) is a scale for assessing adult ADHD symptoms and for recollecting childhood ADHD symptoms [50]. Directly linked to the DSM-IV diagnostic criteria, it is available in two forms: long and Quick-Screen. The long form is a self-report scale, which provides scores for the major dimensions of ADHD: Inattention (9 items), Hyperactivity (5 items), and Impulsivity (4 items), plus another dimension, the Sluggish Cognitive Time (SCT) (9 items not present in the Childhood Symptoms versions). Another 3 items are included for evaluating the frequency of symptom occurrence, the age of onset, and the affected areas (home, education, work, and social relationships). The Quick-Screen can be used as a tool to quickly identify the probability of an individual having ADHD; it also includes both self-report and other-report forms (for example, spouse, parent, or sibling) for Current Symptoms (8 items) and Childhood Symptoms (6 items). It does not measure the SCT. The scoring sheets for both versions (long and Quick-Screen) allow you to calculate the percentile corresponding to the score obtained by the subject for each domain and the total score [31,50].

(3) The Diagnostic Interview for ADHD in Adults (DIVA-5) [24], Italian Version DIVA, is a semi-structured interview tool administered by a clinician. DIVA-5 was translated into Italian by Stefano Pallanti and Luana Salerno, at the Clinical Neurosciences Onlus Research Centre (CNS), Florence (Italy). The back translation was done by Michelle Klopper (DIVA foundation). DIVA-5 consists of 3 parts concerning the following areas: (1) ADHD symptoms in childhood and adulthood, (2) age of ADHD onset, and (3) areas of impairment due to ADHD symptoms. It is divided into three parts: the first part analyses 9 symptoms of inattention (A1), the second part analyses 9 symptoms of hyperactivity/impulsivity (A2), and the third part focuses on identifying the areas where the symptomatology causes an alteration of personal, social, and/or work functioning. After having verified the presence of the symptom in adulthood (present in the last 6 months or for a longer period of time), the possible presence of the same during childhood/pre-adolescence is investigated (age considered between 5 and 12 years); the questions investigating the symptoms are accompanied by a series of examples of everyday life situations related to the symptom. The test administration lasts approximately 60–90 min [24,31].

### 2.4. Statistical Analysis

A descriptive statistical analysis was applied to the demographic and clinical variables of our sample, using the mean ± standard deviation (SD) for continuous variables. After having ascertained the normality of distribution by applying the Shapiro–Wilk test, a *t*-test was used for continuous variables with normal distribution and the non-parametric Kruskal–Wallis Χ_2_ test for those with non-normal distribution. Percentages, the Pearson Χ_2_ test, and the Fisher exact test were used for categorical data. The level of statistical probability was set at *p* < 0.05.

The internal consistency of the Italian version of DIVA-5 and BAARS-IV was evaluated applying the Cronbach alpha coefficient [61] and the Kuder coefficient [62] for dichotomous items.

The construct, concurrent, and predictive validity of the Italian version of DIVA-5 scale were analyzed. For the construct validity, we performed an exploratory factor analysis (EFA). The conventional approach of EFA followed by orthogonal (varimax) rotation [63,64] and oblique rotation was used. The factors highlighted by orthogonal rotation were selected according to the Kaiser’s criterion: eigenvalue >1 for each factor [65], subsequently confirmed by the graphical confirmation of the scree plot. Items with factor loadings >0.40 on a given factor were therefore identified as valid indicators of the factor. We analyzed the eigenvalue of each factor, which accounts for the variance of single factor, and the variance communality, defined as the part of variance explained by all factors. To verify the adequacy of the analysis, oblique rotation of the factors was performed, which allowed us to evaluate the interdependence of the factors themselves. The Kaiser–Meyer–Olkin (KMO) measure was used to verify the adequacy of the sample for the application of a factor analysis [66], and Bartlett’s test of sphericity was used to verify the applicability of a factor analysis in our sample [67].

The appropriate sample size for the factor analysis was evaluated through the “rule of 5” in the subjects–variables ratio (“rule of thumb”), which suggests that the number of observations must be equivalent to at least 5 times the number of variables considered [68].

The concurrent validity of the DIVA-5 scale was investigated by evaluating the correlation with the other two administered scales (ASRS-v1.1, BAARS-IV) through the Spearman’s coefficient.

The predictive validity was investigated by applying the stepwise model (forward and backward) of a multiple linear regression between the total score of the DIVA-5 scale (dependent variable) and the selected socio-demographic and clinical variables representing the clinical outcomes.

Finally, we calculated the sensitivity, specificity, and positive and negative predictive values of the DIVA-5 scale in comparison with the BAARS-IV scale, empirically considered the “gold standard” since it has already been validated. With the data obtained, for each dimension (inattention in childhood and adulthood, and inattention and hyperactivity–impulsivity in childhood and adulthood), we created an ROC (Receiver Operating Characteristic) curve, which is a graph that correlates the sensitivity and specificity of a diagnostic test to the variation of the cut-off value. If the AUC (Area Under the Curve) has a value between 0.5 and 1, the test is increasingly more accurate. A *p* value of 0.05 or less was considered statistically significant. All the data were analyzed using the STATA Version 12 software (StataCorp LCC, College Station, TX, USA).

### 2.5. Ethical Considerations

This study was performed in accordance with the ethical standards laid down in the Helsinki II Declaration about informed consent and anonymity.

This study was approved by the Ethics Committee of the Emilia Nord Vast Area (Protocol no. AU00228833/23 of 27 July 2023) and authorized by the Health Directorate of the AUSL-Modena (Decision no. 1696 of 31 July 2023).

All the data were reported in an Excel database. Each participant was given an anonymized number code in order to avoid personal identification. Only the researchers involved in this study had access to the data. All the participants gave their informed consent before participating in this study.

## 3. Results

### 3.1. Socio-Demographic and Clinical Characteristics of Our Sample

Our sample is represented by 132 individuals, distributed homogeneously by sex (50.76% males and 49.24% females). We found no statistically significant differences in all the selected socio-demographic variables between the males and females in our sample (Table 1). Most of the subjects were Italian (87.12%), more than half of the sample had obtained a lower secondary school diploma (57.58%) and were single (59.85%), and approximately half of our sample lived with their origin family and were employed (Table 1).

Regarding the clinical variables in childhood (Table 2), we did not appreciate any statistically significant difference between males and females: the majority of the subjects in our sample did not have any history of family disorders (58.02%) nor socio-relational issues (48.85%); in the cases in which these anamnestic elements were present, the most common family history was a psychiatric disorder (34.35%) and the most frequent issue was represented by parent separation (19.85%). The majority of our sample was not treated at Child Neuropsychiatry Service during childhood and adolescence (68.70%), did not take any psychopharmacological therapies (86.26%), and had no medical or psychiatric comorbidities (64.89%); in the few subjects who had a comorbidity in childhood, this was mainly represented by substance abuse (69.72%), in particular cannabinoids. Consistently, most subjects had not been admitted to a psychiatric ward during childhood (85.27%).

Regarding the clinical variables in adulthood (Table 3), 56.78% of the subjects were treated in one outpatient service, mainly at the Mental Health Centre (MHC), and the prevalent psychiatric comorbidity was represented by a personality disorder (18.18%), in particular, borderline personality disorder. The prescription of pharmacological ADHD therapy statistically significantly differed between the two sexes (Pearson Χ_2_ = 11.23; *p* = 0.05). In particular, no drug therapy prescription was more frequent among females compared to males (33.33% vs. 26.02%; RS > 2; *p* < 0.05). In 23.58% of the cases, the most frequent pharmacological ADHD drug prescribed was represented by atomoxetine (Table 3).

**Figure 1:**
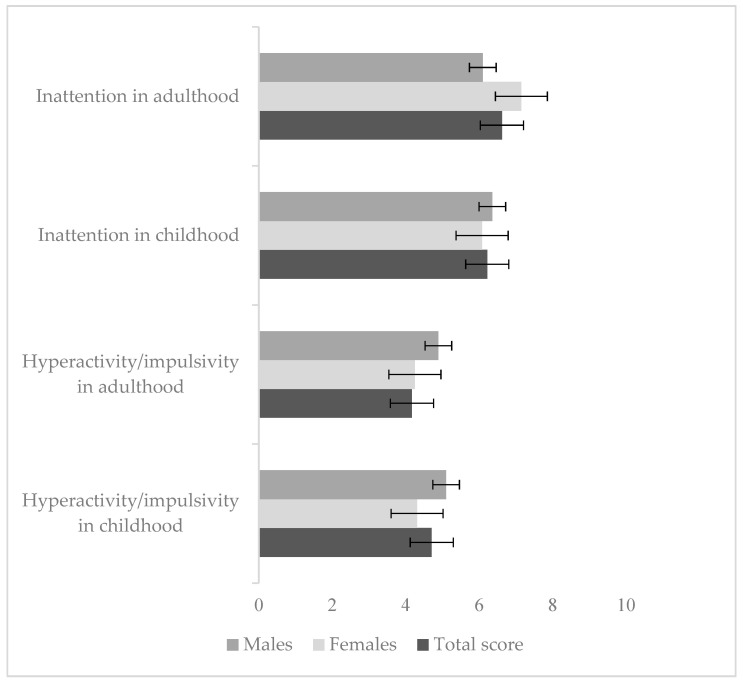
DIVA-5 score (means ± standard deviation).

Our sample obtained the following mean scores for DIVA-5: 6.62 for inattention in adulthood, 6.22 for inattention in childhood, 4.17 for hyperactivity/impulsivity in adulthood, and 4.71 for hyperactivity/impulsivity in childhood, as shown in Figure 1. Females obtained higher scores than males for current inattention (7.15 vs. 6.10), with a reversed score for hyperactivity/impulsivity in childhood (4.31 vs. 5.10), without any statistically significant difference between the two sexes.

**Figure 2:**
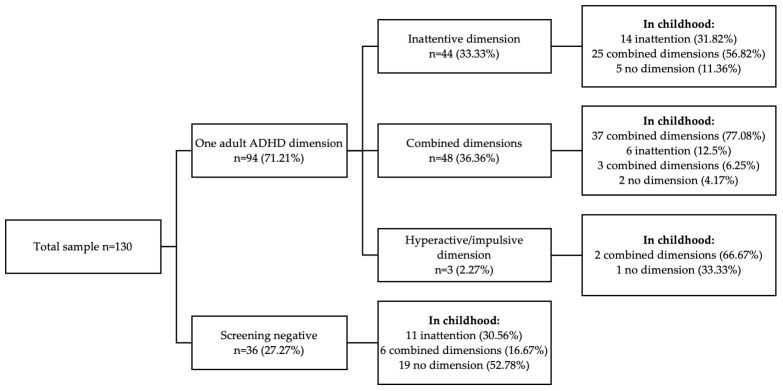
DIVA-5 screening of inattentive and hyperactive/impulsive dimensions in adulthood and childhood.

Based on the scores obtained for the adult dimensions of the DIVA-5 scale, one current ADHD dimension was present in the majority of cases (*n* = 94; 71.21%). Both the inattentive and hyperactive/impulsive dimensions were present in 36.36% (*n* = 48) of our sample, only one inattentive dimension (*n* = 44) in 33.33%, and one hyperactive/impulsive dimension in 2.27% (*n* = 3) (Figure 2). If we analyze the cases with a DIVA-5 score suggesting inattention in adulthood (*n* = 44), we see that 56.82% (*n* = 25) of them showed both inattention and hyperactivity/impulsivity in childhood, 27.27% (*n* = 14) showed only attention deficit, whereas 11.36% (*n* = 5) did not show any ADHD symptoms in childhood (Figure 2). The subjects who presented current combined ADHD deficit at the DIVA-5 (*n* = 48) presented the following characteristics in childhood: 77.08% (*n* = 37) both ADHD dimensions, 12.5% (*n* = 6) only attention deficit, and 6.25% (*n* = 3) hyperactivity/impulsivity, whereas 4.17% (*n* = 2) did not present any ADHD symptoms in childhood (Figure 2). Only three individuals of our sample presented current hyperactivity/impulsivity at the DIVA-5 scale: two of them (66.67%) already showed similar alteration in both dimensions in minor age, whereas one (33.33%) did not present any ADHD alteration in childhood (Figure 2). In our sample, the inattentive dimension was present in 92 individuals (69.70%), whereas hyperactivity/impulsivity was present in only 51 individuals (38.64%) (Figure 2). Among the subjects with negative adult ADHD symptoms at our DIVA-5 screening (*n* = 36; 27.27%), 16.67% (*n* = 6) of them showed alteration in both ADHD dimensions in childhood, 30.56% (*n* = 11) only an attention deficit, whereas 52.78% showed no altered dimensions in childhood (Figure 2).

Regarding the persistence of ADHD symptoms from childhood to adulthood according to the DIVA-5 scores, we observed that inattention, which was presented in 78.79% of the sample in childhood (*n* = 104), was maintained in 88.46% of the adult cases (*n* = 92), whereas the dimension of hyperactivity/impulsivity, which was presented in 58% of the child cases (*n* = 77), was maintained only in 49.35% of the adult subjects (*n* = 38).

The ASRS-v1.1 mean score obtained by the subjects in our sample was 4.78 out of a maximum score of 6; females reported a higher mean score (4.98) than males (4.57) in a statistically significant way (Kruskal–Wallis Χ_2_ = 3.849, *p* = 0.0498) (Table 4).

**Figure 3:**
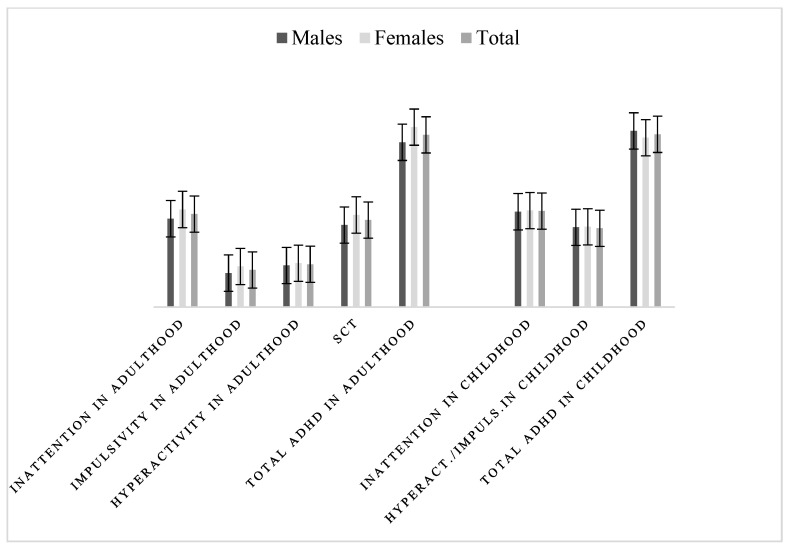
BAARS-IV score (means ± standard deviation).

The BAARS-IV scale scores are reported in Figure 3 and in Table 4: the average score of adulthood inattention was 27.10 and childhood inattention was 27.98, with higher scores among female subjects (28.46 vs. 25.78 in adulthood and 28.14 vs. 27.82 in childhood), with a statistically significant difference between childhood and adulthood only in the adult attention dimension (Kruskal–Wallis Χ_2_ = 5.696; *p* = 0.0170). We find statistically significant differences between the two sexes in the impulsivity in adulthood (Kruskal–Wallis Χ_2_ = 11.763; *p* = 0.0006, score) and in the total score of the BAARS-IV scale in adulthood (Kruskal–Wallis Χ_2_ = 6.494; *p* = 0.0108). The BAARS-IV scale, differently from the others, also evaluates the dimension of Sluggish Cognitive Time (SCT) in adulthood. Our sample had an average SCT score of 25.36, with a statistically significant higher score in females than males (Kruskal–Wallis Χ_2_ = 6.638, *p* = 0.0100). The average BAARS-IV total score of ADHD was 50.21 in adulthood and 50.36 in childhood.

### 3.2. Reliability Analysis of the ASRS-V1.1, DIVA-5, and BAARS-IV

The internal consistency of the DIVA-5 scale was confirmed by Cronbach’s alpha value 0.77 for inattention in adulthood, 0.61 for hyperactivity/impulsivity in adulthood, 0.77 for inattention in childhood, and 0.76 for hyperactivity/impulsivity in childhood. The Kuder coefficient was 0.77 for inattention in adulthood, 0.62 for hyperactivity/impulsivity in adulthood, 0.78 for inattention in childhood, and 0.77 for hyperactivity/impulsivity in childhood. Both the coefficients ranged between 0.62 and 0.78, indicating the acceptable reliability of the scale [69].

### 3.3. Concurrent Validity Analysis of the DIVA-5

The DIVA-5 scores of inattention in both adulthood and childhood positively correlated with the ASRS-v1.1 score, as well as the hyperactivity/impulsivity dimension score in adulthood. Conversely, childhood hyperactivity/impulsivity was not correlated with the ASRS-v1.1 score (Table 5). A positive statistically significant correlation between the dimensions of attention deficit in adulthood and childhood evaluated through the DIVA-5 and the BAARS IV can be noted as well as between the dimensions of hyperactivity/impulsivity in adulthood and childhood. Finally, it is interesting to note how the SCT score positively correlated with the adult inattentive and negatively with the childhood hyperactivity/impulsivity score of the DIVA-5 (Table 5).

### 3.4. Factor Analysis of the DIVA-5 Scale

**Figure 4:**
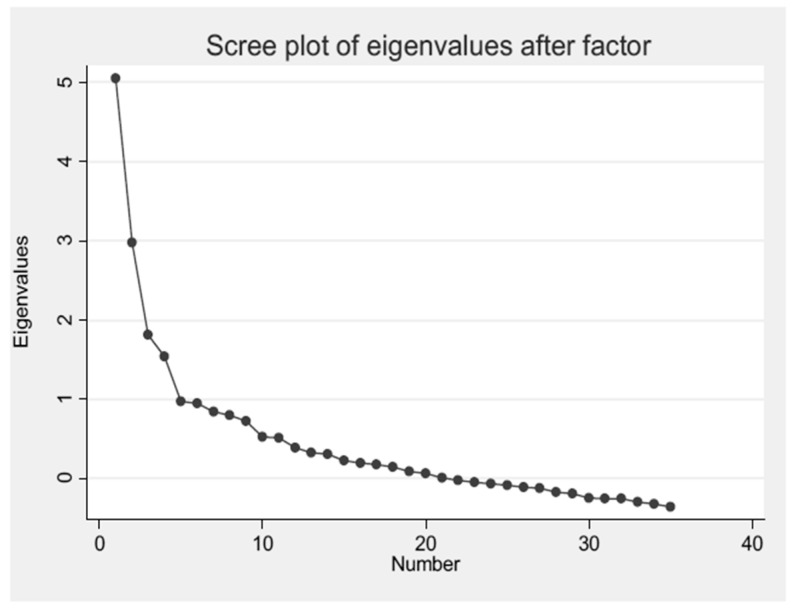
Scree plot of exploratory factor analysis (EFA).

The exploratory factor analysis highlighted five factors with an eigenvalue > 1 as shown in the scree plot (Figure 4).

**Figure 5:**
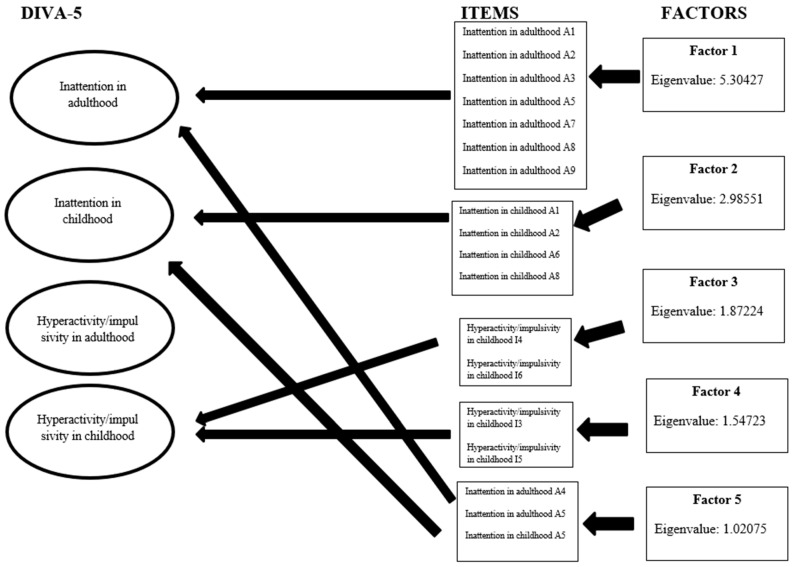
Distribution of factors in ADHD dimensions.

The composition of the factors was obtained by performing an orthogonal rotation that allowed us to highlight the items that underlie the five factors as shown in Figure 5.

In Table 6, the eigenvalues of each factor and the DIVA-5 items which underlay each factor are shown. Based on the values of uniqueness, it was highlighted that items A1, A9, I/I3, and I/I 5 were those with a lower uniqueness and therefore a greater specific variance (Table 6).

The oblique rotation substantially confirmed the orthogonal one, highlighting the same five main factors, positively correlated with each other (promax matrix); the correlation matrix of the five rotated factors gave us information on how much the factors were not correlated and therefore how important they were in providing specific information. Factor 2 presented the highest value of the oblique rotation matrix ranged between 0.4600 and 0.7383, which explained the variance in our sample in a higher percentage between 46% and 73% than the other factors (Table 7).

The numerical adequacy of the sample for factor analysis was evaluated through the Kaiser–Meyer–Olkin test (KMO), which obtained a value of 0.69. According to Bartlett’s test of sphericity, which was statistically significant (Χ_2_ = 1506.34; *p* < 0.001), the correlation matrix between the various items was adequate.

### 3.5. Predictive Validity Analysis

We applied two stepwise forward and backward models of multiple linear regression analyses. The first one between the DIVA-5 scale score (dependent variable) and the sociodemographic variables (independent variables) highlighted that “work activity” was associated with the DIVA-5 total score in a statistically significant way (coeff. 0.932; 95% CI: 0.28; 1.58; *p* = 0.005). In particular, the student status (coeff. 3.192; 95% CI: 0.64; 5.74; *p* = 0.015) was positively correlated with the total score of the DIVA-5 scale in a statistically significant way.

The second one between the DIVA-5 scale total score (dependent variable) and the clinical variables (independent variables) showed that the variable statistically significantly associated in a negative way was the therapy for ADHD (coeff. −1.147; 95% CI: −1.98; −0.31; *p* = 0.008), specifically, the variable “no therapy “ (coeff. −6.885; 95% CI: −11.64; −2.13; *p* = 0.005).

### 3.6. Sensitivity and Specificity Analysis of the DIVA-5 Scale

The specificity and sensitivity values of the four dimensions of the DIVA-5 compared to the respective dimensions of the BAARS-IV, which we consider the reference scale as it has already been validated, are shown in Table 8. The DIVA-5 presented the highest sensitivity to identify attention deficit in adulthood and the highest specificity in detecting hyperactivity/impulsivity in childhood, with the highest positive predictive value in the attention deficit dimension in childhood and the highest negative predictive value in attention deficit in adulthood. The highest prevalence value was related to attention deficit in childhood. The sensitivity of the attention dimension in adulthood and childhood presented values ranged between 83.47 and 98.76%, with a specificity ranged between 69.23 and 72.73%; for the hyperactive/impulsive dimension in adults and children, however, the DIVA-5 scale showed a sensitivity between 47.76% and 68.93% and a specificity between 76% and 82.14%.

For the ROC curves (Figures S1–S4), we found an AUC value of 0.7071 for adult and 0.6670 for child inattentive dimensions, respectively, whereas for the dimensions of hyperactivity and impulsivity in childhood and adulthood, the values of the respective AUCs were 0.6762 and 0.5740.

## 4. Discussion

### 4.1. Characteristics of Our Sample

More than 30% of our subjects were still studying. This finding is in line with the literature reporting that persistent ADHD in young adulthood is associated with the prolongation of study years and lower academic performance compared to peers [70]. We found a similar percentage of students (30.30%) in the Korean study on the validity of the DIVA-5 (29.7%) [55]. The prevalent family in history of pathologies was psychiatric and more than 50% of our sample presented early negative life experiences, especially divorce of parents and socio-economic issues, in line with the ADHD literature [71,72].

Most subjects in our sample were not treated and cared for by the Child Neuro Psychiatry Service, supporting the hypothesis that some adult ADHD cases, diagnosed as so-called “late onset”, are cases of adult subjects not being adequately diagnosed and treated during childhood [24,73]. The most frequent comorbidity was substance use, especially cannabinoids, consistently with the tendency of early toxicophilia in subjects with ADHD [74,75]. This greater vulnerability would be secondary to the search for a stimulus that increases the brain level of dopamine in a sort of “self-medication” [75,76].

Most of our sample was treated by at least one mental health service/professional in adulthood, a fact already noted by other authors [77]. We can infer this result from the negative conditioning determined by ADHD on normal neurodevelopment, especially if ADHD is not recognized and treated during childhood. Personality disorder, in particular borderline disorder (70.83%), was the psychiatric diagnosis most frequently presented in comorbidity with ADHD, suggesting the overlap between some characteristics of cluster B personality disorders, particularly borderline, and the dimensions of impulsivity and emotional dysregulation in ADHD [78].

### 4.2. The DIVA-5, BAARS, and ASRS Scores

In our sample, the most prevalent adult ADHD type was the combined type (36.92%), followed by the inattentive type (33.33%) and hyperactive/impulsive type (2.27%).

From the total score of the DIVA-5 scale, we found a percentage of individuals with symptoms suggestive of ADHD in adulthood equal to 71.21%. The type of ADHD with combined presentation was the most frequent in adults, followed by the inattentive one, while the alteration in the hyperactivity/impulsivity domain alone was poorly represented according to the DIVA-5 score. The data of our sample are partially superimposable to those obtained for the validation of the DIVA-5 in Farsi [56], in which only 38% of the participants had ADHD according to the DIVA-5 score.

Our data highlighted a greater persistence of the inattention symptom in adulthood compared to the hyperactive/impulsive (88.46% vs. 49.35%), perfectly aligned with all the data in the literature on adult ADHD, that consider attention deficit a fundamental dimension of this disorder in adulthood [72]. At the same time, we observed a reduction in the hyperactivity/impulsivity dimension during adulthood, also in line with the literature [79], suggesting that the impulsivity/hyperactivity dimension more specifically represents the core of the neurodevelopmental disorder that undergoes normalization during the growth of the individual.

We found that the attention deficit was present only in adulthood in a few subjects of our sample (11.36%), as reported by some authors [28]. These results can be interpreted differently: they may represent participants’ difficulty in remembering their childhood conditions, or they may indicate an unrecognized or subthreshold ADHD suffered during childhood [73]. Alternatively, they may indicate an additional “adult-only” subtype of ADHD, which should be explored further [80].

Regarding the inattentive dimension, our results from the scores on the DIVA-5 and BAARS-IV highlight that it is more frequent in the female sex, totally in accordance with the literature [11,81]. This result is also consistent with the higher scores in the Sluggish Cognitive Time dimension of the BAARS-IV in females, which could represent an extreme of attention deficit with a paradoxical “slowing down” of the cognitive faculties rather than the hyperactivity/impulsivity characteristic of ADHD [50]. Clinical data relating to ADHD hyperactive/impulsive dimension show the prevalence of this dimension in the male population in accordance with the scientific literature [82,83], except for the BAARS-IV score for this dimension which was positive among the females in our sample. This result could be interpreted by a more sensitive perception of this symptom in the female population since BAARS-IV is a self-administrated scale.

Similarly, the different administration methods, self-administered for the BAARS-IV scale and hetero-administered for the DIVA-5 scale, could have influenced the lower number of subjects with positive symptoms for ADHD on the DIVA-5 compared to the BAARS-IV in the two dimensions both in childhood and in adulthood, since the subjective assessment of ADHD symptoms can be overestimated, as reported by some authors [84].

### 4.3. Validation Analysis of the DIVA-5

This is the first study in the literature evaluating the validity of the Italian version of the DIVA-5, to our knowledge.

The reliability analysis of the DIVA-5 showed an acceptable internal consistency with Cronbach’s alpha values and Kuder’s coefficient in the Italian version. Regarding the concurrent validity, a statistically significant correlation was highlighted for both the ASRS-v1.1 and the BAARS-IV, suggesting a good reliability of DIVA-5 to diagnose ADHD dimensions. It should also be underlined that the DIVA-5 scale identified a smaller number of subjects with positive ADHD symptoms compared to the BAARS scale, suggesting greater specificity and lower sensitivity, as indicated by other authors [54,56].

Our factor analysis highlighted five factors that appear to be equally distributed between the inattentive dimension in childhood and adulthood and the hyperactive/impulsive dimension in adults. Factor 1 was supported by specific items for the inattentive dimension of adults, while factor 2 only by items for the child inattentive dimension; factors 3 and 4 were supported by specific items for the child hyperactive/impulsive dimension, and only factor 5 was underpinned by items for the inattentive dimension, in common between childhood and adulthood. This result suggests that the inattentive and hyperactive/impulsive dimensions are specific and poorly overlapping. Factor 2 appears to be the one most representative of the variance of our sample, suggesting that this dimension can represent the core symptom of ADHD. Our factors were not representative of all four dimensions, since our analysis did not find evidence of any factor for the hyperactive/impulsive dimension in adulthood, probably because this dimension was the least represented in our sample.

By applying the multiple linear regression model between the DIVA-5 scale score and the sociodemographic/clinical outcomes, we highlighted a statistically significant association related to the work activity of our sample and to the specific pharmacological therapy for ADHD; specifically, the highest scores on the DIVA-5 would correspond to the categories of “student” and “no therapy in progress”. These results, which are similar to what was found in the Korean study [55], suggest the delay in completing formal education compared to peers, a fact highlighted by other authors [85,86] and which confirms the predictive validity of the scale regarding any social criticalities and need for therapeutic interventions in adult ADHD [7].

Finally, our analyses showed that the DIVA-5 scale is more sensitive for the detection of attention deficit, both in adulthood and in childhood, and is more specific for the hyperactivity/impulsivity dimension, both in adults and children. The AUC values for all the DIVA-5 dimensions were >0.5, suggesting a good accuracy of the DIVA-5. Although the Korean and Persian studies used different gold standards in their analyses (ASRS-v1.1 for Korean and clinical diagnosis for Persian), both studies rated the DIVA-5 as superior in sensitivity, specificity, PPV, and NPV, as in our study. However, we should highlight that our study is the only one that used a test similar in length and items for comparison with the DIVA-5 [55,56].

### 4.4. Limitation and Advantages

Our study shows some limitations:

- The size of our sample, which, although it was adequate for the analyses we performed, represents a potentially limiting element because it is not sufficiently representative of the general population;
- The internal consistency of the Italian version of DIVA-5 was only acceptable: reliability tests, including test–retest, inter- and intra-rater reliability, as well as the cross-cultural test, were not applied;
- The monocentric design, which does not allow the complete generalization of the results;
- The intrinsic difference in the nature of the scales used which can expose to operator-dependent bias;
- The lack of a universally recognized gold standard and a structured interview for the diagnosis of ADHD in adults, which can further limit our results.

However, our study has the advantage of having demonstrated the potential usefulness of the DIVA-5 scale in identifying the symptoms of ADHD in adulthood, suggesting its good applicability in daily clinical practice for diagnostic purposes. Given the clinical difficulty in identifying reliable and accurate indicators to diagnose ADHD in the absence of biological markers, scale validation studies allow for refining the diagnosis in order to identify patients who can benefit from appropriate pharmacological and non-pharmacological treatments. Moreover, scale validation studies could assist in providing data for better-informed decision-making in clinical settings and drug research and development. A further merit is that of having deepened the knowledge in the clinical area of ADHD in adulthood, still little-known today.

## 5. Conclusions

In conclusion, our study highlights that the Italian version of the DIVA-5 can be considered a valid and reliable tool, like the versions already validated in other languages, for adult ADHD diagnosis. Our analysis underscores the acceptable reliability and good validity of the Italian version of DIVA-5 in detecting both inattentive and hyperactive/impulsive dimensions in childhood and adulthood. These two dimensions proved to be independent in our exploratory factor analysis and with different expressions in the life stages, showing a transition from hyperactivity/impulsive in childhood to inattentiveness in adulthood.

The implementation of DIVA-5 will allow Italian clinicians to observe the characteristics of the subject with ADHD from a dimensional perspective, rather than simply detecting the presence/absence of the pathology, providing useful information on the most suitable therapeutic and rehabilitation choice for everyone. Especially for adult ADHD, which represents a disorder still little studied and explored today, the need to have psychometric tools to support the clinician takes on considerable importance both for clinical treatment and for research purposes. Finally, we hope to have contributed to deepening the knowledge on ADHD, the interest in the study of which is shared not only by the scientific community, but also by the general population, since, thanks also to the use of social media, curiosity about ADHD in adulthood has been a growing trend in recent years.

## Figures and Tables

**Table 1:** Socio-demographic variables of our sample. ^a^ Kruskal–Wallis Χ_2_. SD: standard deviation.

Socio-Demographic Variables	Males	Females	Total Score	Pearson Χ_2_	*p* Value
Sex, n (%)	67 (50.76%)	65 (49.24%)	132 (100%)		
Age (ys), mean ± SD	29.24 ± 8.82	29.72 ± 10.08	29.48 ± 9.43	0.045 ^a^	0.83
Nationality, n (%)				1.26	0.53
Italian	57 (43.18%)	58 (43.94%)	115 (87.12%)		
European non-Italian	5 (3.79%)	2 (1.52%)	7 (5.30%)		
Non-European	5 (3.79%)	5 (3.79%)	10 (7.58%)		
Marital status, n (%)				0.46	0.79
Single	42 (31.82%)	37 (28.03%)	79 (59.85%)		
Engaged/married	24 (18.18%)	27 (20.45%)	51 (38.64%)		
Widowed/divorced	1 (0.76%)	1 (0.76%)	2 (1.52%)		
Education, n (%)				6.52	0.09
Degree	1 (0.76%)	1 (0.76%)	2 (1.52%)		
High school	17 (12.88%)	12 (9.09%)	29 (21.97%)		
Middle school	42 (31.82%)	34 (25.76%)	76 (57.58%)		
Primary school	7 (5.30%)	18 (13.64%)	25 (18.94%)		
Employment, n (%)				5.34	0.25
Employee	33 (25.00%)	27 (20.45%)	60 (45.45%)		
Freelancer	1 (0.76%)	5 (3.79%)	6 (4.55%)		
Unemployed	12 (9.09%)	12 (9.09%)	24 (18.18%)		
Retired	2 (1.52%)	0 (0%)	2 (1.52%)		
Student	19 (14.39%)	21 (15.91%)	40 (30.30%)		
Living condition, n (%)				2.06	0.56
Alone	9 (6.87%)	15 (11.45%)	24 (18.32%)		
Family of origin	36 (27.48%)	30 (22.90%)	66 (50.38%)		
Acquired family	20 (15.27%)	19 (14.50%)	39 (29.77%)		
Protected structure	1 (0.76%)	1 (0.76%)	2 (1.52%)		
Homeless	0 (0%)	0 (0%)	0 (0%)		

**Table 2:** Clinical variables of our sample in childhood.

Clinical Variables	Males	Females	Total Score	Pearson Χ_2_	*p* Value
Family history of pathologies, n (%)				3.11	0.54
Psychiatric	18 (13.74%)	27 (20.61%)	45 (34.35%)		
Substance use	3 (2.29%)	3 (2.29%)	6 (4.58%)		
Other illnesses	1 (0.76%)	1 (0.76%)	2 (1.53%)		
None	43 (32.82%)	33 (25.19%)	76 (58.02%)		
Socio-relational-familial issues, n (%)				7.34	0.29
Adoption	4 (3.05%)	0 (0%)	4 (3.05%)		
Mourning of relative	4 (3.05%)	5 (3.82%)	9 (6.87%)		
Divorce of parents	11 (8.40%)	15 (11.45%)	26 (19.85%)		
Illness	2 (1.53%)	3 (2.29%)	5 (3.82%)		
Socio-economic	5 (3.82%)	6 (4.58%)	11 (8.40%)		
Others	4 (3.05%)	8 (6.11%)	12 (9.16%)		
None	36 (27.48%)	28 (21.37%)	64 (48.85%)		
Psychiatric treatments, n (%)				0.26	0.61
Yes	22 (16.79%)	19 (14.50%)	41 (31.30%)		
No	44 (33.59%)	46 (35.11%)	90 (68.70%)		
Pharmacological therapy, n (%)				8.21	0.22
Methylphenidate/atomoxetine	4 (3.05%)	0 (0%)	4 (3.05%)		
Antipsychotics	2 (1.53%)	0 (0%)	2 (1.53%)		
Antidepressants	2 (1.53%)	1 (0.76%)	3 (2.29%)		
Mood stabilizers	1 (0.76%)	0 (0%)	1 (0.76%)		
Benzodiazepines	1 (0.76%)	1 (0.76%)	2 (1.53%)		
More than one kind	2 (1.53%)	4 (3.05%)	6 (4.58%)		
None	54 (41.22%)	59 (45.04%)	113 (86.26%)		
Comorbidities, n (%)				7.03	0.32
Substance use	41 (33.60%)	44 (36.07%)	85(64.89%)		
Psychiatric	16 (13.11%)	11 (9.02%)	27 (22.13%)		
Neurodevelopmental	0 (0%)	1 (0.82%)	1 (0.82%)		
Neurological	1 (0.82%)	1 (0.82%)	2 (1.64%)		
Medical	0 (0%)	3 (2.46%)	3 (2.46%)		
None	2 (1.64%)	0 (0%)	2 (1.64%)		
More than one	1 (0.82%)	0 (0%)	1 (0.82%)		
Previous psychiatric hospitalizations, n (%)				0.02	0.89
Yes	10 (7.57%)	12 (9.09%)	22 (16.67%)		
No	56 (43.41%)	54 (41.86%)	110 (85.27%)		

**Table 3:** Clinical variables of our sample in adulthood.

Clinical Variables	Males	Females	Total	Pearson Χ_2_	*p* Value
Psychiatric comorbidity (ICD-9-CM), n (%)				4.23	0.75
Schizophrenia spectrum	3 (2.27%)	3 (2.27%)	6 (4.55%)		
Mood disorders	3 (2.27%)	4 (3.03%)	7 (5.30%)		
Personality disorders	9 (6.82%)	15 (11.36%)	24 (18.18%)		
Anxiety disorders	9 (6.82%)	5 (3.79%)	14 (10.61%)		
Psycho-organic disorders	1 (0.76%)	1 (0.76%)	2 (1.53%)		
Substance abuse	0 (0%)	1 (0.76%)	1 (0.76%)		
Social Maladjustment	1 (0.76%)	3 (2.27%)	4 (3.03%)		
Other	1 (0.76%)	1 (0.76%)	2 (1.53%)		
None	44 (32.84%)	33 (24.63%)	77 (57.46%)		
Treatment and care, n (%)					
MHC	35 (29.66%)	32 (27.12%)	67 (56.78%)	6.28	0.28
Psychology	2 (1.69%)	1 (0.85%)	3 (2.54%)		
Private specialist	0 (0%)	4 (3.39%)	4 (3.39%)		
SUS	9 (7.63%)	9 (7.63%)	18 (15.25%)		
More than one	4 (3.39%)	1 (0.85%)	5 (4.24%)		
None	10 (8.47%)	11 (9.32%)	21 (17.80%)		
Psychiatric hospitalization, n (%)				1.59	0.45
Yes	4 (3.03%)	2 (1.52%)	6 (4.55%)		
No	55 (41.67%)	51 (38.64%)	106 (80.30%)		
Unreported	8 (6.06%)	12 (9.09%)	20 (15.15%)		
Socio-relational issues, n (%)				2.90	0.24
Yes	3 (2.36%)	7 (5.51%)	10 (7.87%)		
No	52 (40.94%)	45 (35.43%)	97 (76.38%)		
Unreported	8 (6.30%)	12 (9.45%)	20 (15.75%)		
Medical comorbidities, n (%)				5.78	0.05
Present	2 (1.64%)	8 (6.56%)	10 (8.20%)		
None	54 (44.26%)	41 (33.61%)	95 (77.87%)		
Unreported	7 (5.74%)	10 (8.20%)	17 (13.93%)		
Substance use, n (%)				6.80	0.24
Cannabis	8 (6.25%)	7 (5.47%)	15 (11.72%)		
Cocaine	3 (2.34%)	0 (0%)	3 (2.34%)		
Alcohol	1 (0.78%)	0 (0%)	1 (0.78%)		
Sedatives	1 (0.78%)	0 (0%)	1 (0.78%)		
None	45 (35.16%)	48 (37.5%)	93 (72.66%)		
Unspecified	5 (3.91%)	10 (7.81%)	15 (11.72%)		
Prescribed drug therapy, n (%)				11.23	0.05
Methylphenidate	6 (4.88%)	1 (0.81%)	7 (5.69%)		
Atomoxetine	19 (15.45%)	10 (8.13%)	29 (23.58%)		
Bupropion	4 (3.25%)	3 (2.44%)	7 (5.69%)		
Fluoxetine	1 (0.81%)	4 (3.25%)	5 (4.07%)		
Other	2 (1.63%)	0 (0%)	2 (1.63%)		
None	32 (26.02%)	41 (33.33%)	73 (59.35%)		

**Table 4:** ASRS-v1.1 and BAARS-IV score. CI: confidence interval; SD: standard deviation.

ASRS Score		Mean	SD	CI	Kruskal–Wallis Χ_2_ and *p* Value
Males		4.57	1.16	4.28; 4.86	Χ_2_ = 3.85 *p* = 0.0498
Females		4.98	0.85	4.77; 5.20	
Total		4.78	1.03	4.59; 4.96	
**BAARS—IV score**		**Mean**	**SD**	**CI**	**Kruskal–Wallis Χ_2_ and *p* value**
Adult inattention	Males	25.78	6.08	24.29; 27.26	Χ_2_ = 5.696 *p* = 0.0170
	Females	28.46	3.95	27.48; 29.44	
	Total	27.10	5.29	26.19; 28.01	
Adult hyperactivity	Males	12.13	3.76	11.22; 13.05	Χ_2_ = 1.162 *p* = 0.2810
	Females	12.80	3.66	11.89; 13.71	
	Total	12.46	3.71	11.82; 13.10	
Adult impulsivity	Males	9.91	3.16	9.14; 10.68	Χ_2_ = 11.763 *p* = 0.0006
	Females	11.82	2.93	11.09; 12.54	
	Total	10.85	3.18	10.30; 11.40	
Sluggish Cognitive Time (SCT)	Males	23.90	6.62	22.28; 25.51	Χ_2_ = 6.638 *p* = 0.0100
	Females	26.86	4.61	25.72; 28.00	
	Total	25.36	5.89	24.34; 26.37	
Total adult ADHD	Males	48.03	10.52	45.46; 50.60	Χ_2_ = 6.494 *p* = 0.0108
	Females	52.46	9.39	50.13; 54.79	
	Total	50.21	10.19	48.46; 51.97	
Child inattention	Males	27.82	5.84	26.40; 29.24	Χ_2_ = 0.736 *p* = 0.3910
	Females	28.14	7.50	26.28; 30.00	
	Total	27.98	6.68	26.83; 29.13	
Child hyperactivity/impulsivity	Males	23.27	6.72	21.62; 24.92	Χ_2_ = 0.094 *p* = 0.7592
	Females	23.40	7.38	21.57; 25.22	
	Total	22.99	7.02	22.12; 24.55	
Total	Males	51.33	11.17	48.59; 54.08	Χ_2_ = 0.012 *p* = 0.9139
	Females	49.37	15.50	45.53; 53.21	
	Total	50.36	13.47	48.03; 52.69	

**Table 5:** Correlations between the DIVA-5, the BAARS-IV, and the ASRS v1.1 scores.

	DIVA-5 Adult Inattention Spearman’s Coeff.; *p* Value	DIVA-5 Child Inattention Spearman’s Coeff.; *p* Value	DIVA-5 Adult Hyperactivity/Impulsivity Spearman’s Coeff.; *p* Value	DIVA-5 Child Hyperactivity/Impulsivity Spearman’s Coeff.; *p* Value
BAARS—IV Adult inattention	0.4782; *p* = 0.0000	0.1454; *p* = 0.0963	0.1159; *p* = 0.1857	−0.0772; *p* = 0.3789
BAARS—IV Child inattention	0.3194; *p* = 0.0002	0.5407; *p* = 0.0000	0.0432; *p* = 0.6227	0.3186; *p* = 0.0002
BAARS—IV Adult hyperactivity	0.2656; *p* = 0.0210	0.1574; *p* = 0.0714	0.4766; *p* = 0.0000	0.1886; *p* = 0.0303
BAARS—IV Adult impulsivity	0.3646; *p* = 0.0000	0.0647; *p* = 0.4613	0.4553; *p* = 0.0000	0.0816; *p* = 0.3524
BAARS—IV Child hyperactivity/impulsivity	0.3439; *p* = 0.0001	0.4683; *p* = 0.0000	0.3781; *p* = 0.0000	0.6121; *p* = 0.0000
Sluggish Cognitive Time (SCT)	0.3335; *p* = 0.0001	0.0153; *p* = 0.8620	−0.0387; *p* = 0.6595	−0.1910; *p* = 0.0283
ASRS-v1.1	0.3487; *p* = 0.0001	0.2459; *p* = 0.0055	0.3494; *p* = 0.0001	0.0828; *p* = 0.3566

**Table 6:** Items underlying the factors highlighted in EFA.

DIVA-5				
Inattention Items	Adulthood		Childhood	
	Factor and Factor Loadings (FL)	Uniqueness	Factor and Factor Loadings (FL)	Uniqueness
A1	FACTOR 1 FL: 0.7434	0.3569	FACTOR 2 FL: 0.7119	0.3321
A2	FACTOR 1 FL: 0.6099	0.4255	FACTOR 2 FL: 0.6326	0.3821
A3	FACTOR 1 FL: 0.4019	0.5399		0.4721
A4	FACTOR 5 FL: 0.6987	0.4637		0.4843
A5	FACTOR 1 FL: 0.4391 FACTOR 5 FL: 0.4458	0.4371	FACTOR 5 FL: 0.5151	0.5017
A6		0.6221	FACTOR 2 FL: 0.6836	0.4147
A7	FACTOR 1 FL: 0.4103	0.4574		0.4724
A8	FACTOR 1 FL: 0.6655	0.4515	FACTOR 2 FL: 0.4183	0.5199
A9	FACTOR 1	0.3339		0.3355
**Hyperactivity/impulsivity items**	**Adulthood**		**Childhood**	
	**Factor and Factor Loadings (FL)**	**Uniqueness**	**Factor and Factor Loadings (FL)**	**Uniqueness**
I/I1	FACTOR 6 FL: 0.0006	0.4756		0.5213
I/I2		0.4856		0.4756
I/I3		0.6023	FACTOR 4 FL: 0.7745	0.3198
I/I4		0.5635	FACTOR 3 FL: 0.6886	0.3637
I/I5		0.5304	FACTOR 4 FL: 0.6016	0.3407
I/I6		0.5354	FACTOR 3 FL: 0.5583	0.5072
I/I7		0.5372		0.3984
I/I8	FACTOR 6 FL: 0.6457	0.4752		0.5124
I/I9		0.4659		0.4828

**Table 7:** Oblique rotation matrix of factor analysis.

	Factor 1	Factor 2	Factor 3	Factor 4	Factor 5
Factor 1	−0.3020	0.7618	−0.0418	−0.0625	−0.5103
Factor 2	0.7267	0.5271	0.7383	0.5796	0.5136
Factor 3	0.2014	−0.2040	−0.2965	0.0624	0.1979
Factor 4	−0.0197	−0.1474	−0.1588	0.0935	0.0624
Factor 5	−0.1228	−0.0702	0.2039	−0.2117	−0.1181

**Table 8:** Sensitivity and specificity analysis of the DIVA-5. CI: confidence interval.

DIVA-5	Value (%)	CI min (%)	CI max (%)
Adult inattention			
Sensitivity	98.76	85.82	95.70
Specificity	69.23	61.36	77.10
Positive predictive value	96.43	93.26	99.59
Negative predictive value	45.00	36.51	53.49
Prevalence	90.15	85.07	95.23
Child inattention			
Sensitivity	83.47	77.13	89.81
Specificity	72.73	66.13	80.32
Positive predictive value	97.12	94.26	99.97
Negative predictive value	28.57	20.86	36.28
Prevalence	91.67	86.95	96.38
Adult hyperactivity/impulsivity			
Sensitivity	47.76	39.14	56.18
Specificity	76.00	68.71	83.29
Positive predictive value	89.47	84.24	94.71
Negative predictive value	25.33	17.91	32.75
Prevalence	81.06	74.38	87.74
Child hyperactivity/impulsivity			
Sensitivity	68.93	61.01	66.86
Specificity	82.14	75.58	88.70
Positive predictive value	93.42	89.18	97.77
Negative predictive value	41.82	33.37	50.26
Prevalence	70.63	71.61	85.65

## Data Availability

The original contributions presented in this study are included in the article/Supplementary Materials; further inquiries can be directed to the corresponding author, but due to privacy and ethical restrictions, individual data are unavailable.

## Supplementary Materials

The following supporting information can be downloaded at https://www.mdpi.com/article/10.3390/healthcare13030244/s1: Figure S1: ROC curve of inattentive dimension in adulthood; Figure S2: ROC curve of inattentive dimension in childhood; Figure S3. ROC curve of hyperactive/impulsive dimension in adulthood; and Figure S4. ROC curve of hyperactive/impulsive dimension in childhood.
