# Supplementary material for: Validity of the Italian Version of DIVA-5: Semi-Structured Diagnostic Interview for Adult ADHD Based on the DSM-5 Criteria
Source: Healthcare (Basel). 2025 Jan 26;13(3):244. doi: 10.3390/healthcare13030244 (PMC11816683; doi:10.3390/healthcare13030244)
Supplement: Supplementary file 1 [file healthcare-13-00244-s001.zip › healthcare-3416344-supplementary.pdf]

healthcare

MDPI

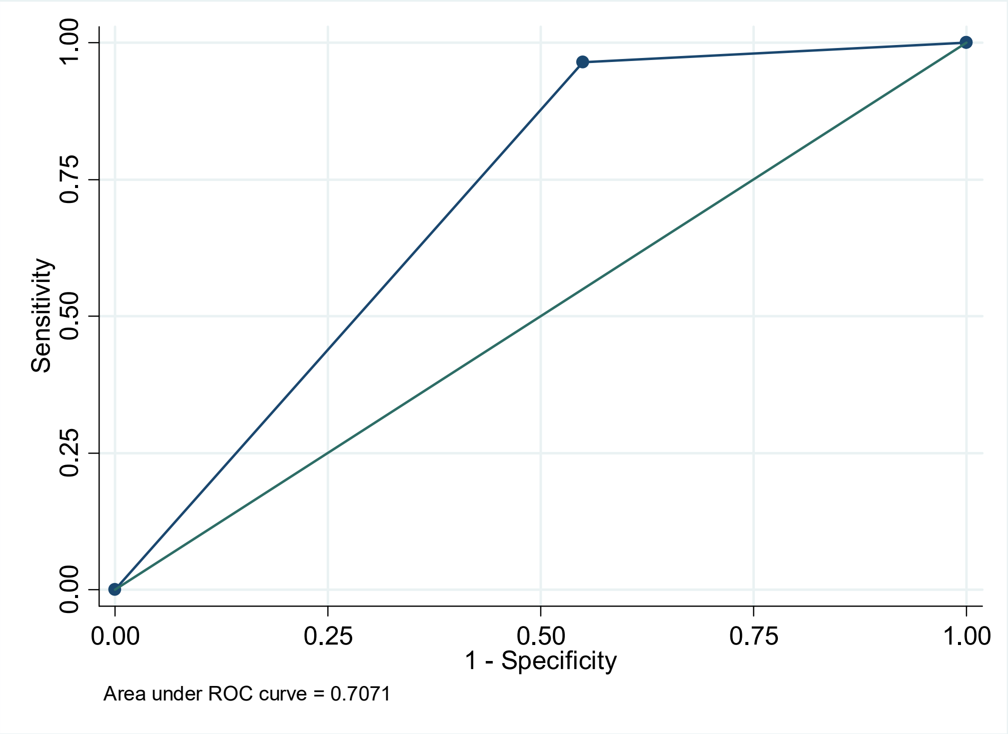

Figure S1. ROC curve of inattentive dimension in adulthood.

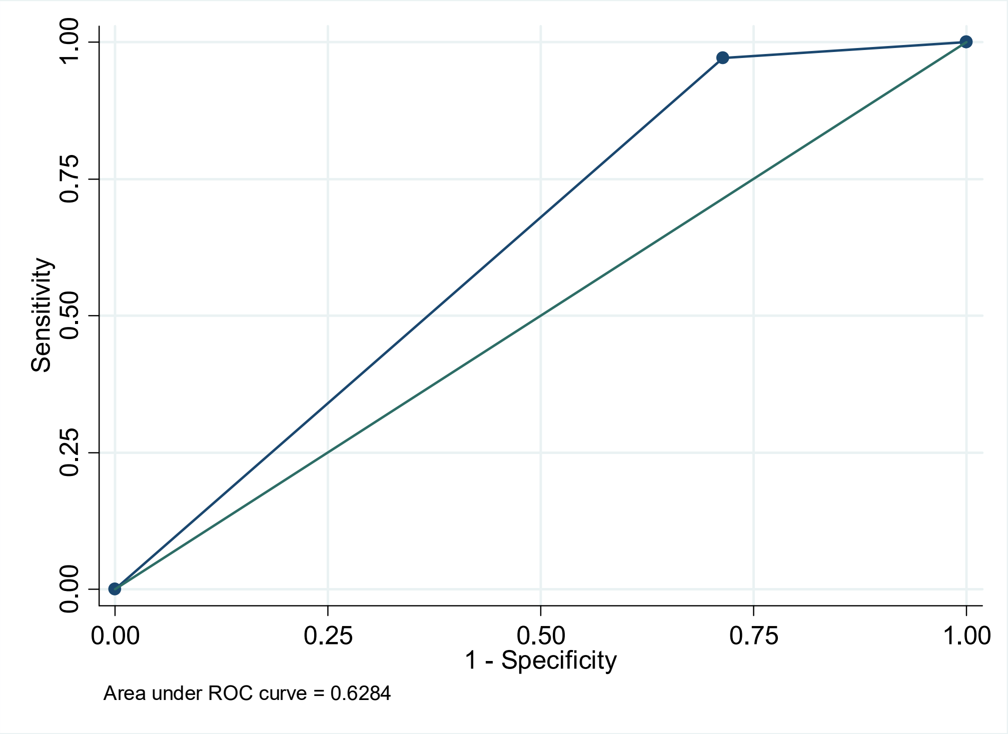

Figure S2. ROC curve of inattentive dimension in childhood.

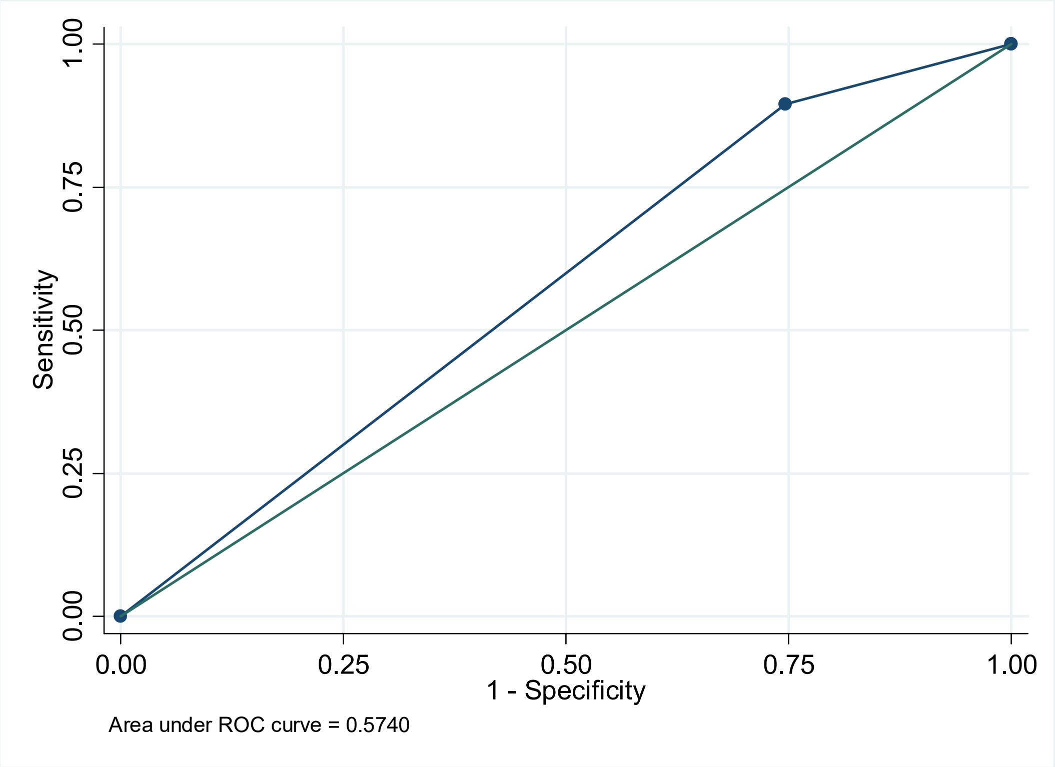

Figure S3. ROC curve of hyperactive/impulsive dimension in adulthood.

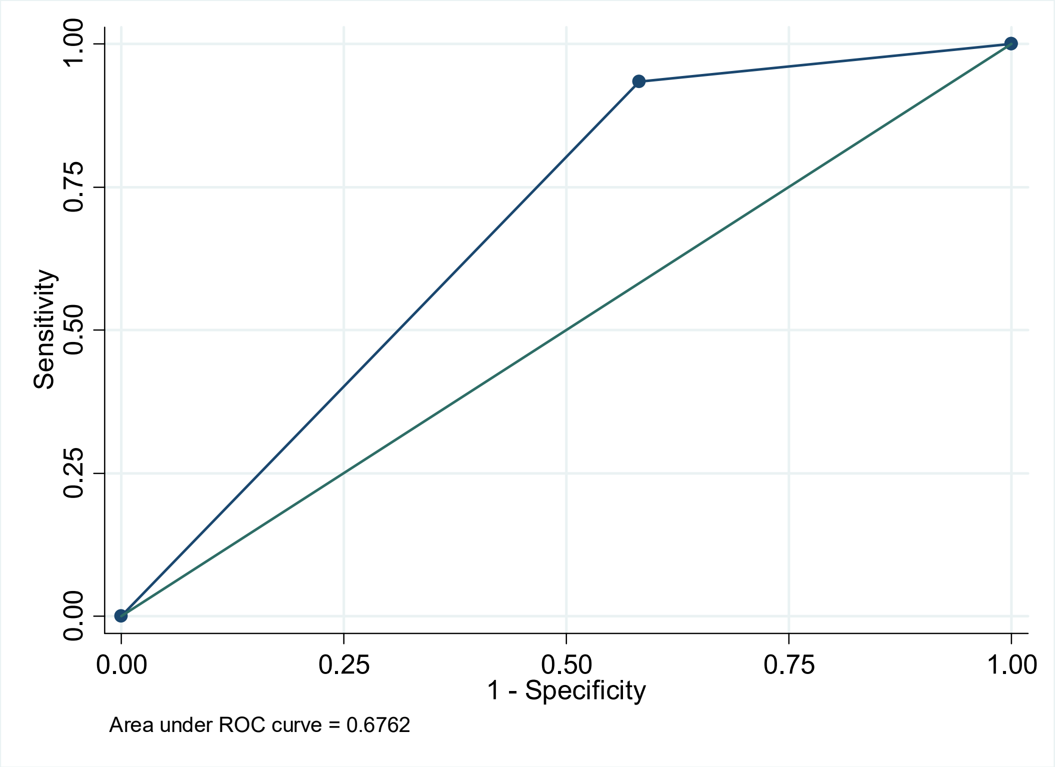

Figure S4. ROC curve of hyperactive/impulsive dimension in childhood.

Disclaimer/Publisher's Note: The statements, opinions and data contained in all publications are solely those of the individual author(s) and contributor(s) and not of MDPI and/or the editor(s). MDPI and/or the editor(s) disclaim responsibility for any injury to people or property resulting from any ideas, methods, instructions or products referred to in the content.
